# Revealing the pathogenesis of gastric intestinal metaplasia based on the mucosoid air-liquid interface

**DOI:** 10.1186/s12967-024-05276-7

**Published:** 2024-05-17

**Authors:** Simeng Liu, Huijuan Wen, Fazhan Li, Xia Xue, Xiangdong Sun, Fuhao Li, Ruoyu Hu, Huayuan Xi, Francesco Boccellato, Thomas F Meyer, Yang Mi, Pengyuan Zheng

**Affiliations:** 1 Henan Key Laboratory of Helicobacter pylori & Microbiota and Gastrointestinal Cancer, Marshall Medical Research Center, The Fifth Affiliated Hospital of Zhengzhou University, No. 3, Kangfuqian Street, Erqi District, Zhengzhou, Henan 450002 China; 2 Department of Gastroenterology, The Fifth Affiliated Hospital of Zhengzhou University, Zhengzhou, Henan 453000 China; 3 Department of Molecular Biology, Max Planck Institute for Infection Biology, 10117 Berlin, Germany; 4 Nuffield Department of Clinical Medicine, Ludwig Institute for Cancer Research, University of Oxford, Oxford, 11743 UK; 5 Laboratory of Infection Oncology, Institute of Clinical Molecular Biology, Christian Albrecht University of Kiel and University Hospital Schleswig-Holstein - Campus Kiel, Rosalind-Franklin- Straße 12, 24105 Kiel, Germany

**Keywords:** Air-liquid interface, Gastric intestinal metaplasia, Markers, mucus secretion, Mucinomic sequencing, Transcriptomic sequencing

## Abstract

**Background:**

Gastric intestinal metaplasia (GIM) is an essential precancerous lesion. Although the reversal of GIM is challenging, it potentially brings a state-to-art strategy for gastric cancer therapeutics (GC). The lack of the appropriate in vitro model limits studies of GIM pathogenesis, which is the issue this work aims to address for further studies.

**Method:**

The air-liquid interface (ALI) model was adopted for the long-term culture of GIM cells in the present work. This study conducted Immunofluorescence (IF), quantitative real-time polymerase chain reaction (qRT-PCR), transcriptomic sequencing, and mucoproteomic sequencing (MS) techniques to identify the pathways for differential expressed genes (DEGs) enrichment among different groups, furthermore, to verify novel biomarkers of GIM cells.

**Result:**

Our study suggests that GIM-ALI model is analog to the innate GIM cells, which thus can be used for mucus collection and drug screening. We found genes *MUC17*, *CDA*, *TRIM15*, *TBX3*, *FLVCR2*, *ONECUT2*, *ACY3*, *NMUR2*, and *MAL2* were highly expressed in GIM cells, while *GLDN*, *SLC5A5*, *MAL*, and *MALAT1* showed down-regulated, which can be used as potential biomarkers for GIM cells. In parallel, these genes that highly expressed in GIM samples were mainly involved in cancer-related pathways, such as the MAPK signal pathway and oxidative phosphorylation signal pathway.

**Conclusion:**

The ALI model is validated for the first time for the in vitro study of GIM. GIM-ALI model is a novel in vitro model that can mimic the tissue micro-environment in GIM patients and further provide an avenue for studying the characteristics of GIM mucus. Our study identified new markers of GIM as well as pathways associated with GIM, which provides outstanding insight for exploring GIM pathogenesis and potentially other related conditions.

**Supplementary Information:**

The online version contains supplementary material available at 10.1186/s12967-024-05276-7.

## Introduction

Gastric intestinal metaplasia (GIM) is a pathological type of intestinal mucosa-like morphological and structural alternations in the gastric mucosa, and represents a risk factor for intestinal-type gastric cancer (GC) [1, 2]. Approximately 0.25% and 10% of GIM patients progress to GC annually in Western Europe and East Asia, respectively [3, 4]. GIM has been recognized as a precancerous lesion and its reverse can be significant for GC curing. GIM can be led by chronic inflammation in stomach, mainly caused by *Helicobacter pylori* infection, followed by autoimmune diseases [5]. However, its pathogenesis remains unclear and it is challenging to study GIM without an appropriate in vitro model. Thus, confirming an appropriate in vitro model that is analog to the tissues from GIM patients is essential to GIM and GC studies and treatments.

Clonal results from human gastric specimens suggest that GIM glands are clonal and can form large clonal plaques through the division of the glands. This process is known as “regional carcinogenesis”, indicating GIM originates from gastric mucosal stem cells [6]. The organoids model and the air-liquid interface (ALI) model are two main stem cell models [7], notably, organoids are miniature cell clusters formed by cells in a three-dimensional (3D) space in vitro, which can self-proliferate and differentiate into different cell types [8]. A necessary condition for a successful in vitro model for human disease is the persistent proliferation of certain cells [9]. It has been reported that Wnt and R-spondin administrated in vitro can maintain the stemness of stem cells and promote their continuous proliferation [10, 11]. The previous studies have shown that human gastric mucosal epithelial stem cells can be expanded indefinitely in 3D stromal cultures [12, 13]. The unique advantage of 3D organoids lies in their promising ability to faithfully recapitulate the intricate structure, functional properties, and genetic characteristics of the original tissue in vivo. This feature enables them to serve as a highly realistic and representative model, allowing for the accurate simulation of the complex dynamics observed in GIM tissue [14]. However, one limitation of 3D organoids is their tendency to form compact structures, which can hinder the penetration of drugs or microorganisms into the innermost cells [15]. Besides, the traditional 3D organoid model has certain restrictions when it comes to collecting mucus. The air-liquid interface (ALI) model addresses these limitations, which makes it ideal for culturing mucus-producing stem cells, in this case, GIM [16].

Due to the complexity of stem cell culture and the substantial differences in the composition of the medium required by various cells [17], researchers have only induced GIM-related phenotypes by overexpressing *CDX2* in 3D organoid models at present. This induction method, however, has certain limitations as it predominantly leads to a reduction in the expression of gastric epithelium-related genes and an increase in the expression of intestinal epithelial-related genes [18]. At present, only human primary tracheal [19] and gastric epithelial cells have been successfully cultured on the ALI model [20], no successful cultivated GIM stem cells have been set on the ALI model.

In this study, human GIM cells were first cultured using the ALI model with the presence of Wnt and R-spondin to maintain the stemness of the GIM stem cells. We aim to construct an effective in vitro model of human GIM cells for further studies and applications, set up new ideas and strategies for the target, pathogenesis and related interventions of GIM, and provide an important reference for the early detection and treatment of GIM.

## Materials and methods

### The selection and exclusion criteria for GIM patients

Three participants with recognized GIM features in any part of the stomach, encompassing focal and extensive, complete and incomplete manifestations, were included in this investigation. The patients diagnosed as opportunistic by GIM through symptomatic services or population-based screening were included. GIM was defined by the histological identification of glands exhibiting phenotypic characteristics of the intestine. Three participants were included irrespective of documented *H. pylori* status, as infection may not be detectable once metaplasia occurred. Individuals with familial syndromes, such as autosomal dominant diffuse gastric cancer, were excluded [21]. Detailed patient information can be seen in Supplementary Table 1.

### Source of cells

This study was approved by the Medical Ethics Committee of the Fifth Affiliated Hospital of Zhengzhou University with the approval number of K201811 (Supplementary File 1). Written consent was obtained from the patients with endoscopic diagnosis of GIM before starting this study. One piece of GIM tissue and another piece of surrounding normal gastric tissue were taken from three patients with biopsy forceps. The GIM and normal gastric tissues were equally divided into two sections, one was placed in 4% paraformaldehyde (PFA) for fixation and the other was used for cell culture.

### Separation of cells

Collagen type II was thawed, and the shaker incubator was set as 37℃ for 35 min. The tissue obtained from the gastroscopy was immersed in medium containing 10% fetal bovine serum, and washed twice with cold (4℃) PBS. Then 2 mL of PBS (+ 40 µL penicillin/streptomycin + 8 µL gentamycin/amphotericin B) was added, and the sample and PBS solution were placed in a centrifuge tube. Next, they were kept at room temperature (18–25℃) for 30 min. PBS was discarded, 3 mL of collagenase type II solution was added to the tube, and the centrifuge tube was shaken at 37 °C for 30–40 min. The solution was fully mixed with a pipette, 3–5 mL of the precooled medium above was added, and the mixture was centrifuged at 1200 rpm under 4℃ for 5 min. The supernatant was discarded and 1 mL of cell basic medium was added. The cells containing the cell basic medium were gently aspirated five times with a 1 mL syringe (if there was still agglomeration, a 40 μm cell sieve was applied to remove the tissue block, and the cells were collected by reverse filtration and placed in another 1 mL centrifuge tube). Cells were counted with a common cell counter.

### The medium composition of the GIM-ALI model

The basal medium widely utilized for culturing mammalian cells is Advanced DMEM/F-12 (ADF) medium [22]. To maintain cell stemness, Wnt3a, R-spondin1, and A83-01 were employed [23, 24]. Tissue culture buffer was provided by 4-(2-hydroxyethyl)-1-piperazineethanesulfonic acid [25]. Stem cell development was facilitated by Glutamax, nicotinamide, and human epidermal growth factor (EGF) [26–28]. Stem cells were maintained by B27 and human noggin [29, 30], while N2 promoted their survival [31]. Human fibroblast growth factor (FGF)-10 and gastrin were capable of promoting stem cell proliferation [32, 33]. The ALI medium composition required for culturing GIM stem cells consists of these components in the following proportions (Table 1).

**Table 1:** ALI medium composition

Factor Name	Final concentration
ADF	18.45% V/V
Wnt3a	50% V/V
R-sondin1	25% V/V
4-(2-hydroxyethyl)-1-piperazineethanesulfonic acid	10 mM
Glutamax	1% V/V
B27	2% V/V
nicotinamide	10 mM
N2	1% V/V
human epidermal growth f-actor(EGF)	20 ng/ml
A83-01	1 µM
human fibroblast growth factor(FGF)-10	150 ng/ml
human noggin	150 ng/ml
human gastrin100 µM	10 nM

### Generation of GIM mucosoid cultures

The inserts (Millipore) were put into 24 wells in advance and the pre-cooled collagen gel (Thermo Fischer) compound solution diluted with ddH_2_O was added. 200 µL ALI medium mixed with 200,000-250,000 primary cells was added to each insert, and 500 µL ALI medium combined with 1 µL Y-27,632 factor (Sigma) was added beneath each insert after the solution solidified. The 24-well plate was placed in a constant temperature incubator with 5% CO_2_, 95% humidity and 37℃ for cultivation. The day as the cells were cultured on the insert was defined to be day zero of cell culture. On the third day, the ALI medium above the insert was removed to start the ALI culture. The medium beneath the insert was subsequently changed twice a week while the mucus above the insert was collected (Supplementary video 1).

### Immunofluorescence

4 µM paraffin-embedded sections were placed in an oven at 65 °C for 2 h and then placed in a xylene I dewaxing tank for 15 min, xylene II dewaxing tank for 15 min, anhydrous alcohol I dewaxing tank for 10 min, anhydrous alcohol II dewaxing tank for 10 min, 95% alcohol dewaxing tank for 5 min, 85% alcohol dewaxing tank for 5 min and 75% alcohol dewaxing tank for 5 min. Next, the sections were washed 3 times with 1×PBS solution for 5 min and then immersed in antigen retrieval solution composed of 12 mL antigen retrieval solution and 588 mL H_2_O for 5 min at 85℃ and 20 min at 65℃ successively, all at room temperature (18–25℃) and washed three times with 1×PBS solution for 5 min. GIM cells were circled with an immunohistochemical pen and 50–100 µL of blocking solution was added to each circle for 1–2 h at room temperature (18–25℃). The primary antibody diluted with the blocking solution was added to the circles and left to stand overnight at 4 °C. The slices were balanced at room temperature (18–25℃) for 1 h the next day and washed 3 times with 1×PBST solution for 5 min. The secondary antibody diluted with the blocking solution was added to the circles at room temperature (18–25℃) for 2 h and the slices were washed 5 times for 5 min with 1×PBST solution. Slices were mounted with the mounting medium containing 4’,6-diamidino-2-phenylindole (DAPI). The images were observed and collected under a fluorescence microscope.

### Quantitative real-time PCR (qRT‐PCR)

The entire process of RNA extraction was carried out on ice. The inserts were washed with cold PBS twice, with 200 µL Trizol added to each wash. The cell suspension was aspirated and collected in a 1.5 mL sterile enzyme-free tube after waiting for 20 min. 400 µL of chloroform was added, the tube was shaken vigorously for 15 s, and left at room temperature (18–25℃) for 5 min. The samples were centrifuged at 4℃ and 12,000 rpm for 15 min. The upper aqueous phase was removed and transferred to a new 1.5 mL sterile enzyme-free tube. Isopropanol equal to the volume of upper aqueous phase was added, slowly shaken 15 times and centrifuged at 4℃, 12,000–13,000 rpm for 10 min. The supernatant was discarded and 1 mL of 75% alcohol was added to the tube, gently shaken, and centrifuged for 5 min. The supernatant was discarded, the remaining pellet was dried in air for 5 min, and dissolved 10 µL of diethyl pyrocarbonate (DEPC) water. The absorbance at A260/A280 was detected by Nando drop ND200, and the RIN value of RNA was determined by Agilent Bioanalyzer 4150. The Reverse Transcription Kit (Roche Diagnostics, Indianapolis, IN) was used to generate complementary DNA. qRT-PCR was performed on the Step One Plus Real-Time PCR System (Applied Biosystems, FosterCity, CA) using the SYBR-Green PCR kit (Roche Diagnostics, Indianapolis, I-N). Glyceraldehyde 3-phosphate dehydrogenase (GAPDH) was used as a reference gene. All primers are shown in Supplementary Tables 2, and the 2^−ΔΔCt^ method [34] used made for qRT-PCR analysis.

### Transcriptomic sequencing

The qualified RNA was prepared according to the instructions of the AB clonal transcriptomic analysis Lib Perp Kit to prepare the PE library. Library quality was assessed using an Agilent Bioanalyzer 4150 and the Illumina Novaseq 6000/MGISEQ-T7 sequencing platform was applied for sequencing. HISAT2 software (http://daehwankimlab.github.io/hisat2/) was employed to compare the clean reads obtained by processing the Perl script [35] with the reference genome [36] to obtain mapped reads, and further FPKM values for each gene were calculated in Feature Counts (http://subread.sourceforge.net/).

### Proteomic sequencing

The GIM and normal mucus were extracted and quantitatively analyzed. The samples were separated by the HPLC liquid phase system Easynlc and analyzed using a Q-Exactive mass spectrometer (AGC, Automatic gain control). The target was set as 1e6, maximum IT as 50 ms and dynamic exclusion time as 60 s. The raw data files were analyzed and processed with the mass spectrometer.

### Differentially expressed genes (DEGs) analysis

The processed transcriptomics data were imported into R software 4. 1. 3 [37], and differential analysis was performed by the “DESeq2” package [38]. The screening threshold for DEGs was |log_2_FC|>1 [39] and *P* value < 0.05 [40]. The “pheatmap” package [41] and “ggplot2” package [42] were applied to draw heat maps and bar graphs.

### Differentially expressed proteins (DEPs) analysis

Quantitative analysis with MaxQuant was utilized to ascertain protein abundance while removing anti-library and contaminant proteins [43]. Proteins lacking any recorded features (those with zero counts across all samples) were excluded [44]. The dataset underwent logarithmic transformation and was then subjected to differential expression analysis employing “Limma” package. Differentially expressed proteins (DEPs) were defined based on a significance threshold of *P* value < 0.05 and a fold change (FC) greater than 1 [45]. The relative protein abundance in gastric tissues was depicted through the creation of heatmaps and bar graphs using ggplot2 in R software [46].

### GO and KEGG enrichment analysis and PPI network

Gene Ontology (GO) and Kyoto Encyclopedia of Genes and Genomes (KEGG) enrichment analyses were performed for the up- and down-regulated DEGs. The “clusterProfiler”, “richplot”, and “ggplot2” packages were used for analysis [47, 48]. The GO terms comprised 3 parts: Biological Process (BP), Cellular Component (CC), and Molecular Function (MF). The KEGG database included the systematic analysis, annotation, and visualization of gene functions [49]. The protein-protein interaction (PPI) network of DEGs was constructed by the STRING online website [50]. The PPI network was analyzed by Cytoscape software (version 3.9.0) [51].

### GEO data processing

The GIM datasets GSE60427, GSE60662, GSE106656, and GSE78523 were retrieved from the Gene Expression Omnibus (GEO) database website (www.ncbi.nlm.nih.gov/geo) [52]. Specifically, GSE60427 comprised 7 normal cases and 8 GIM samples; GSE60662 contained 4 normal samples and 4 GIM samples; GSE106656 featured 7 GIM samples; and GSE78523 encompassed 15 normal samples and 16 GIM samples. These datasets consisted of various platform files, namely GPL17077, GPL13497, GPL6244, and GPL18990, housing clinical data of IM patients. ComBat, a classical Bayesian-based analysis method leveraging known batch information, was employed for batch correction on high-throughput data. The “Combat” algorithm within the R package “SVA” (version 3.29.1) was utilized to mitigate batch effects among different GSE datasets [53]. The data normalization was conducted post-batch effect removal, wherein the average value was considered as the expression value for a gene when multiple probes corresponded to it [54]. Finally, Principal Components Analysis (PCA) was employed to validate the results post-removal. To enhance the credibility of the analysis and verify the results, the GIM samples in GSE60427, GSE60662, and GSE78523 were designated as the train group, while those in GSE106656 were defined as the test group.

### Cluster analysis of essential genes

The hub genes identified from the intersection of the DEGs obtained from the GEO datasets and the DEGs gained from the experimental groups were visualized by Venn diagrams [55]. The “ConsensusClusterPlus” package [56] was used to process cluster analysis on the samples from the GEO datasets. More details were shown in the heatmap.

### Statistics

The statistical analysis was conducted using SPSS 23.0 (SPSS, Inc., Chicago, IL, USA), R software (version 4.2.1), and GraphPad Prism 8.0 software (GraphPad, Inc., La Jolla, CA, USA) [57, 58]. Each experiment was repeated 3 times on 3 samples to ensure robustness and reproducibility. For the identification of differential genes between the two groups of samples, The Wilcox test was utilized for statistical comparison. This non-parametric test is suitable for analyzing data that do not meet the assumptions of normality. It helps determine if there are significant differences between groups [59]. The hypergeometric distribution, a discrete distribution suitable for describing the number of sampling instances from a limited total sample pool, where n samples are drawn each time without replacement until a specified type of sample is obtained, was employed. The hypergeometric test was applied to conduct GO and KEGG enrichment analysis on differentially expressed genes (DEGs) [60]. For comparison of the means of two groups of independent samples, the t test was utilized [61]. Numeric expressions of *P* values were provided for each analysis, with statistical significance defined as *P* < 0.05 (*P < 0.05; **P < 0.01; ***P < 0.001; ****P < 0.0001) [62]. To control for type I errors (false positives) resulting from multiple comparisons, the *P* values of genes were corrected using various methods, including Bonferroni [63], Benjamini-Hochberg [64], Holm, Hochberg [65], Hommel [66], and BY [64]. These correction methods adjust the significance threshold to account for the increased chance of obtaining false positives when conducting multiple statistical tests simultaneously. By applying these correction methods, the study aimed to mitigate bias and enhance the reliability of the results.

The raw data, scripts and supplementary materials have been uploaded to https://www.jianguoyun.com/p/DSA9JDwQw5fKDBiEtc4FIAA.

## Results

### Verification of GIM tissue and cells

**Fig. 1:**
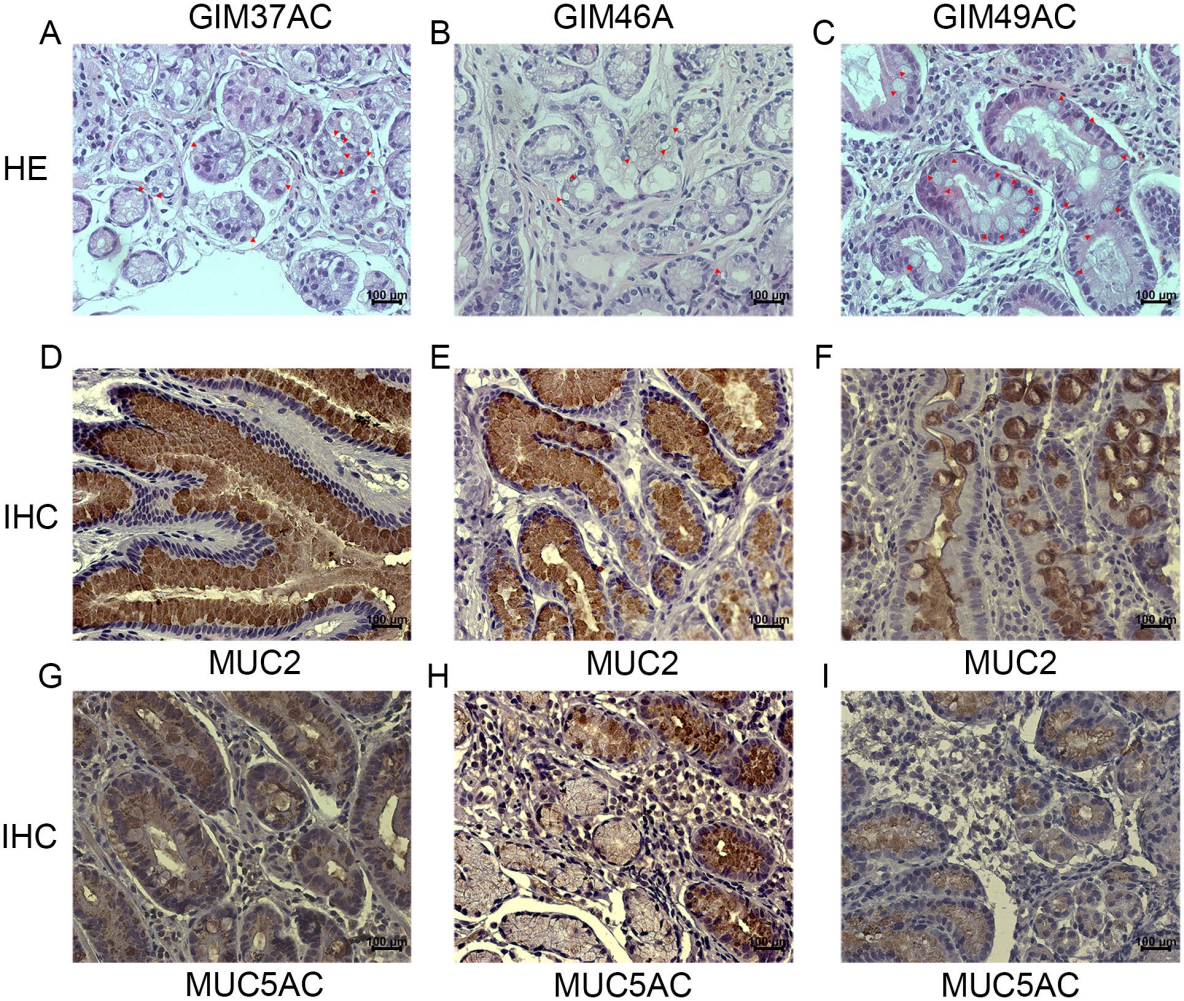
Identification of the GIM organization. (**A**-**C**) HE staining of 3 GIM samples. The nucleus is stained blue, the cytoplasm is stained red and Red arrows indicate goblet cells. (**C**-**F**) Immunohistochemistry of 3 GIM samples. Nuclei are stained blue and *MUC2* is stained brown. (**G**-**I**) Immunohistochemistry of 3 GIM samples. Nuclei are stained blue and *MUC5AC* is stained brown

**Fig. 2:**
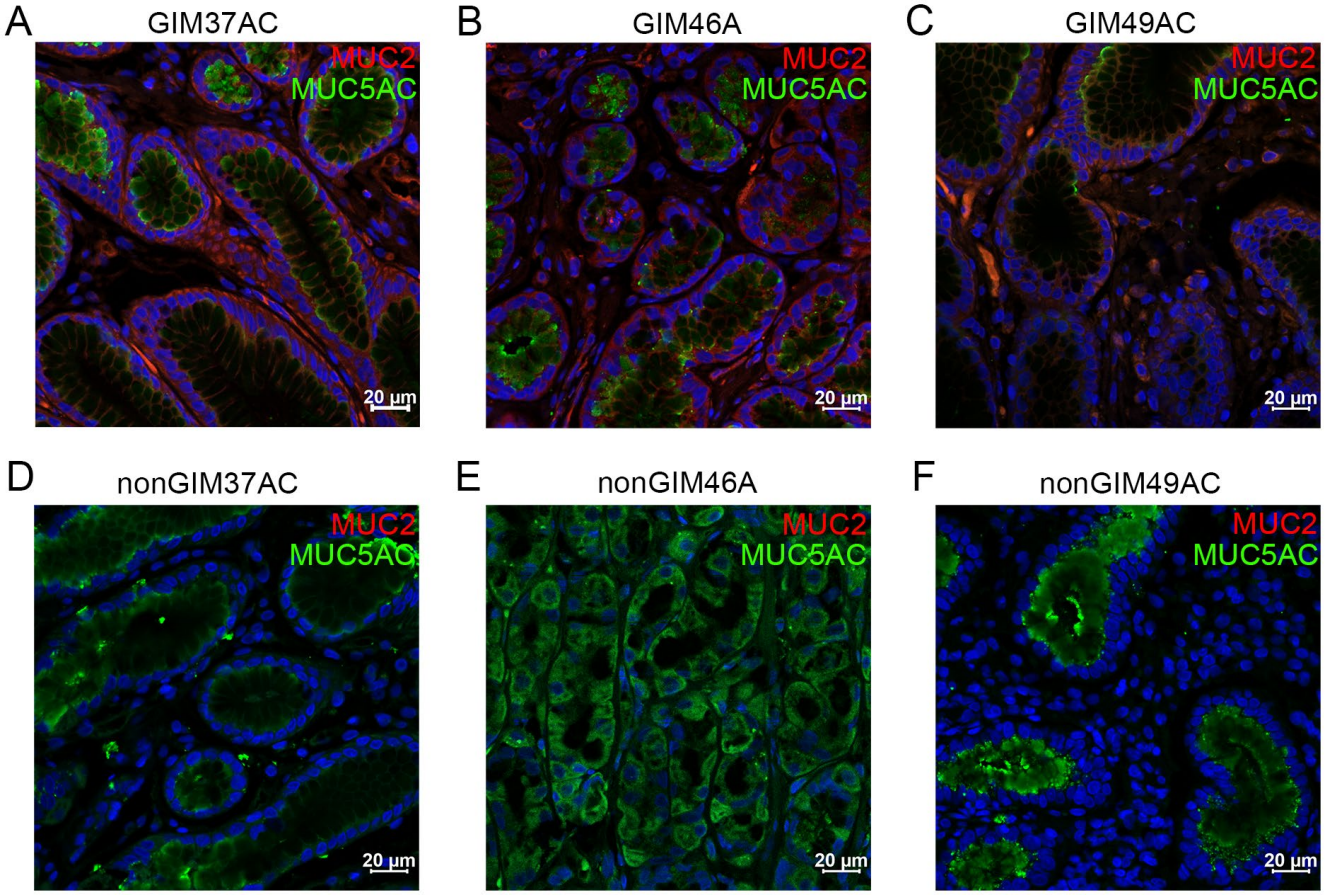
Immunofluorescence of GIM and normal gastric samples. (**A**-**C**) Expression of *MUC5AC* and *MUC2* in GIM samples. (**D**-**F**) The expression of *MUC5AC* and *MUC2* in normal gastric tissue samples. The nucleus is stained blue, *MUC5AC* is stained green, and *MUC2* is stained red

Prominent goblet cells were visible in the GIM tissue after HE staining (Fig. 1A-C). We found that GIM tissue expressed a large amount of *MUC2* and a small amount of *MUC5AC* by immunohistochemistry and immunofluorescence, while normal gastric tissue specifically expressed *MUC5AC* (Figs. 1D-I and 2A-F; Supplementary Fig. 1), which agreed with their molecular characteristics.

**Fig. 3:**
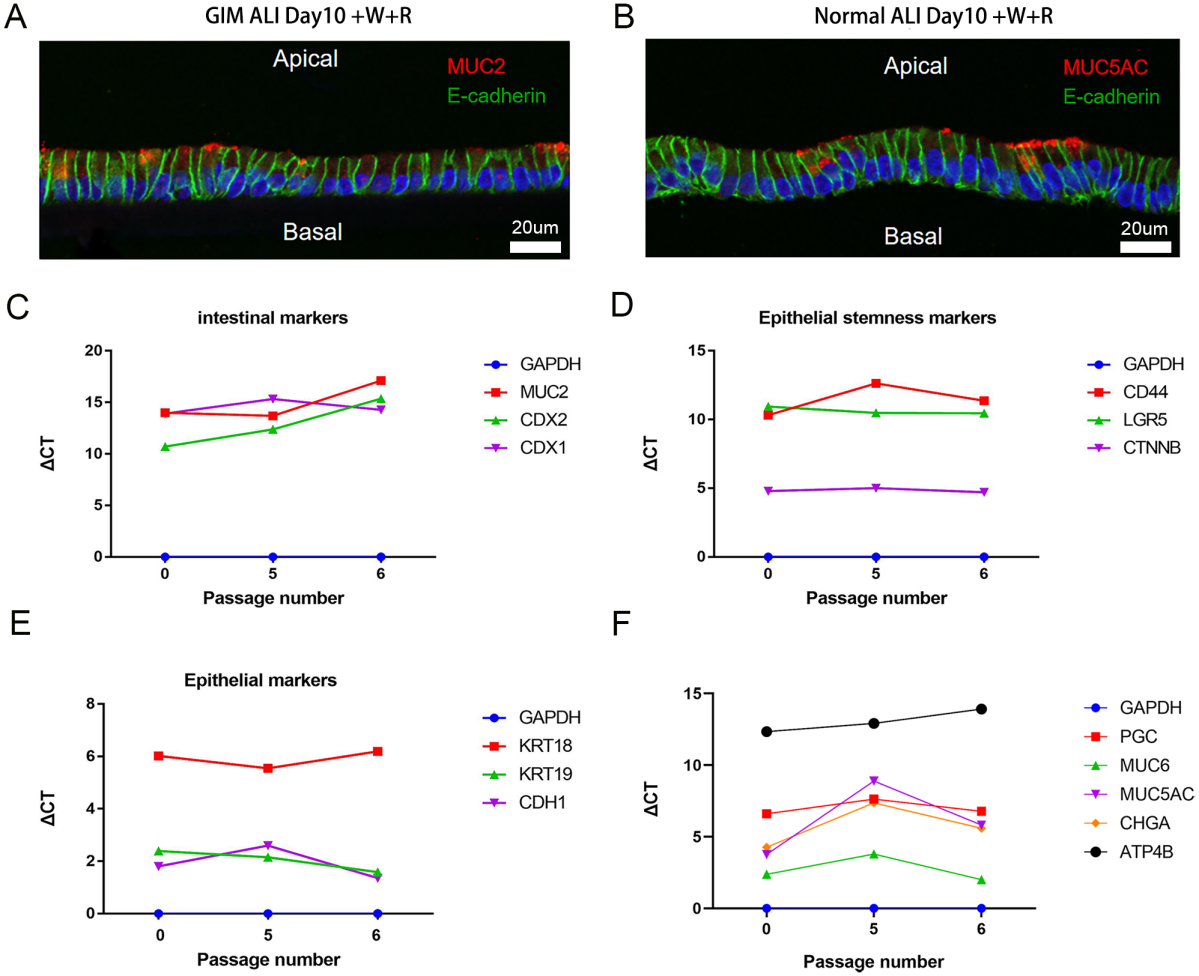
GIM ALI model immunofluorescence and qRT-PCR identification. (**A**) The expression of *MUC2* in GIM samples using the ALI model after 10 days of culture under added Wnt and R-spondin (+ W + R) conditions visualized by red fluorescence (**B**) The expression of *MUC2* in normal samples using the ALI model after 10 days of culture under added Wnt and R-spondin (+ W + R) conditions seen by red fluorescence. (**C**-**F**) Expression of intestinal markers, epithelial stemness markers, epithelial markers and gastric gland cell type markers in GIM samples remain relatively stable with cell passage

We found that GIM and gastric epithelial cells developed into a mature columnar epithelial morphology while GIM and normal samples separately specifically expressed *MUC2* and *MUC5AC* based on immunofluorescence using the ALI model, which is consistent with in vivo findings (Fig. 3A&B; more details in Supplementary Fig. 2). The expression of intestinal markers (*MUC2*, *CDX1*, and *CDX2*), epithelial stemness markers (*CD44*, *LGR5*, and *CTNNB*), epithelial markers (*KRT18*, *KRT19*, and *CDH1*), and gastric gland cell type markers (*PGC*, *MUC6*, *MUC5AC*, *CHGA*, and *ATP4B*) kept relatively stable with the cells’ passage (Fig. 3C-F). Moreover, the importance of each component in the ALI medium was determined by qRT-PCR (Supplementary Fig. 3). These results suggest that our ALI model can be used to study GIM.

### The differentially expressed genes (DEGs) functions between GIM and normal samples

**Fig. 4:**
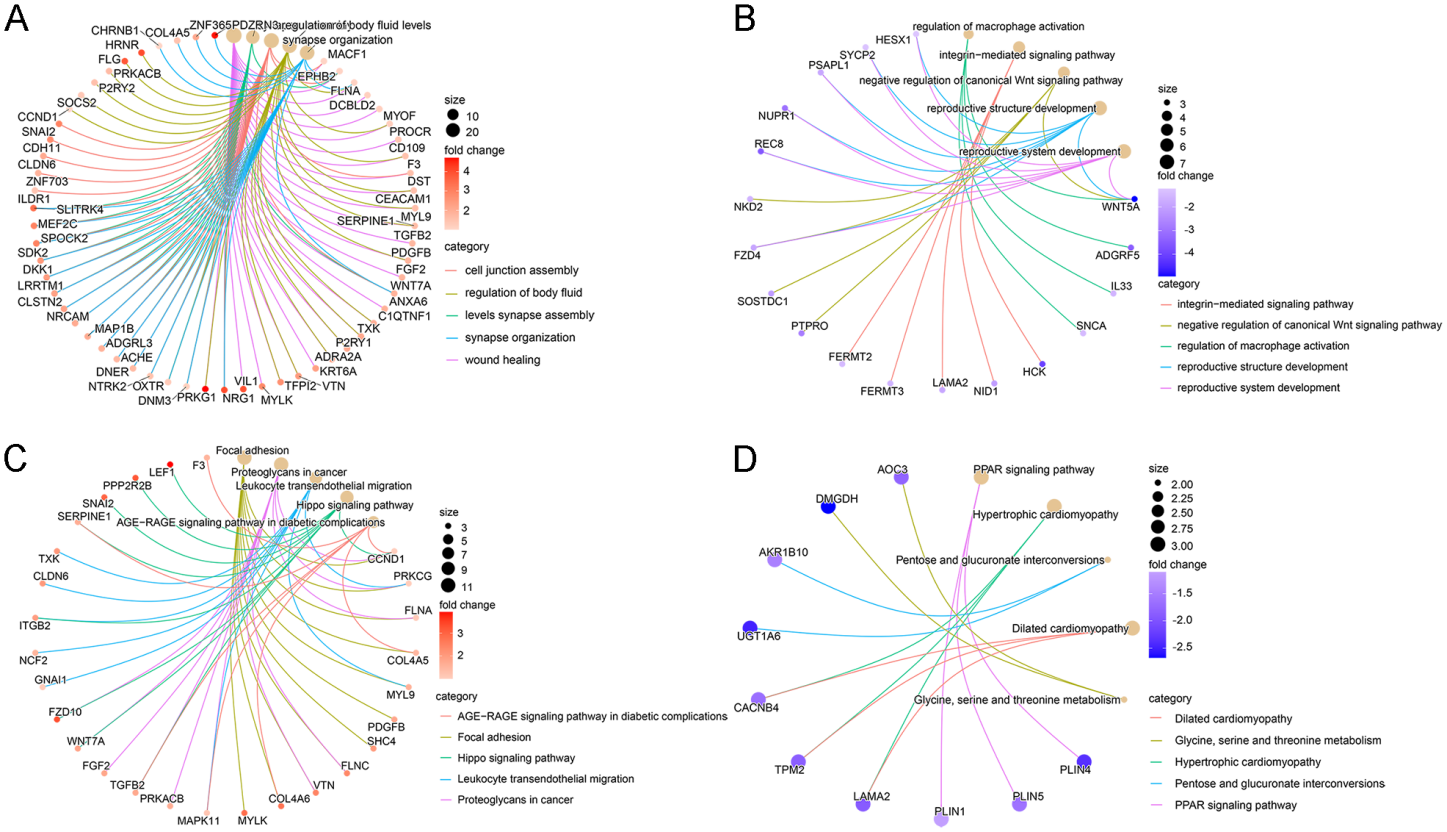
The enrichment and PPI network of DEGs between the GIM and normal group. (**A**-**D**) GO and KEGG pathway enrichment analysis of up-regulated and down-regulated DEGs in GIM samples. Red and purple circles represent up-regulated and down-regulated DEGs in GIM samples, respectively. The larger the circle, the more the DEGs are enriched; the darker the circle, the more significant the difference

To reveal the functions and relationships of DEGs, we conducted enrichment analysis and PPI network analysis. Compared with normal samples, the upregulated DEGs in GIM samples were mainly related to cell junction (Fig. 4A). In contrast, the down-regulated DEGs were primarily enriched in the negative regulation of Wnt signaling pathway by GO enrichment analysis (Fig. 4B). Moreover, the upregulated DEGs were relative to leukocyte transendothelial migration, proteoglycans in cancer (Fig. 4C), while down-regulated DEGs were abundant in the pentose-glucuronic acid conversion pathway and the PPAR lipid synthesis pathway through the KEGG pathway enrichment analysis (Fig. 4D). Eight nodes adjacent to *SEMA5A* and *SPON2* were identified (Supplementary Fig. 4A). The top eight DEGs that were most connected were applied to construct a protein interaction network map (Supplementary Fig. 4B). Multiple pathways enriched in upregulated genes in the GIM samples were associated with GC progression, and these results suggested that GIM may affect GC occurrence through these genes.

### Enrichment analysis of differentially abundant proteins in GIM and normal samples

**Fig. 5:**
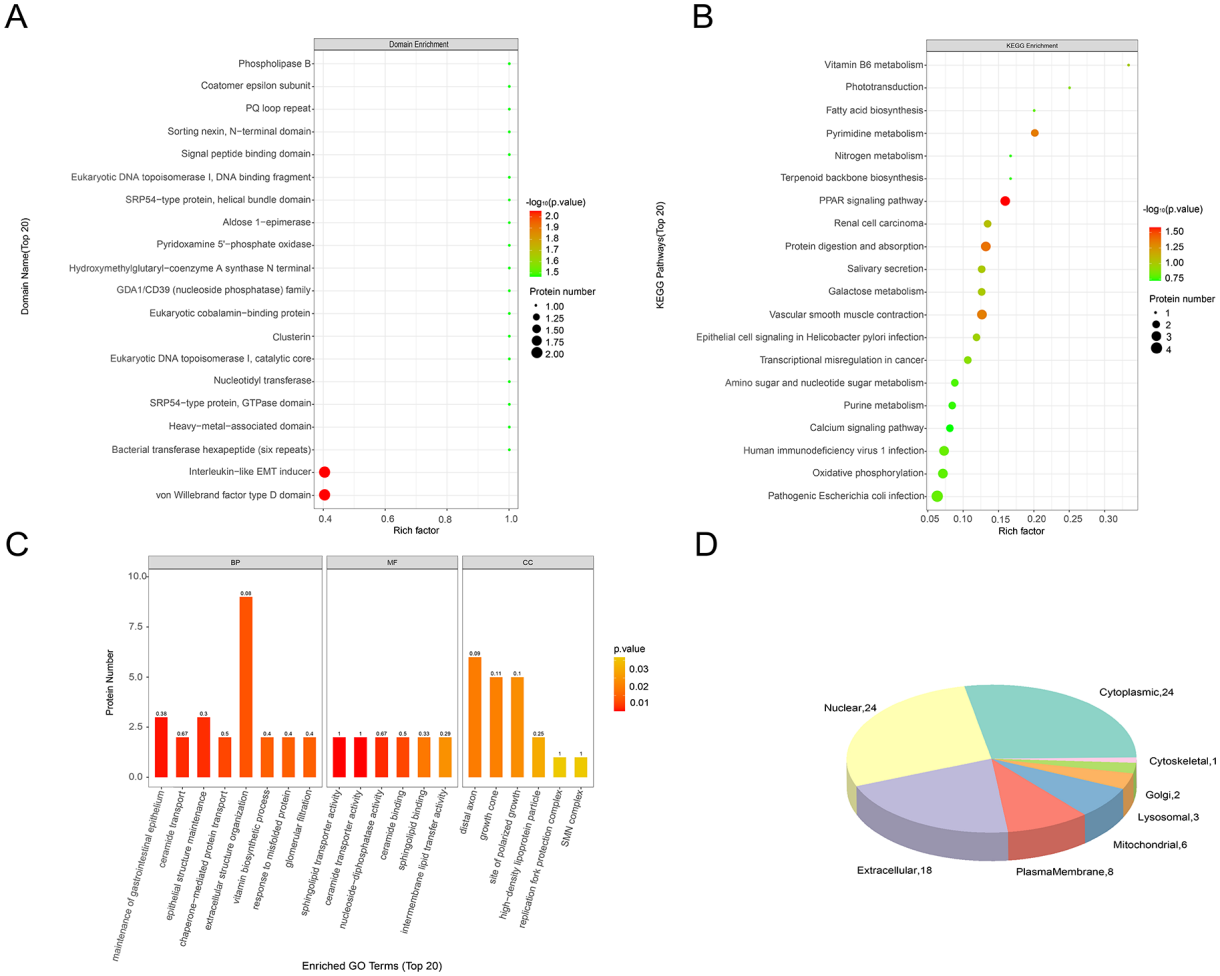
Comparison of differentially abundant proteins between the GIM and normal group. (**A**&**B**) The domain and KEGG pathway enrichment of differentially abundant proteins between GIM and normal samples. The circle size represents the number of differentially abundant proteins enriched in each case: the larger the circle, the higher the number. The color gradually changes from green to red, indicating that the difference is more distinguished. (**C**) The GO enrichment of differentially abundant proteins between the two sample groups. The color gradually changing from yellow to red shows that the difference is becoming more conspicuous. (D) The cellular distribution of differentially abundant proteins between the two sample groups

The enrichment analysis was similarly conducted to learn about the functions of differentially abundant proteins between GIM and normal samples. We found that the differentially abundant proteins were mainly enriched in the interleukin-like epithelial-mesenchymal transition (EMT) inducer domain (Fig. 5A). They were primarily associated with oxidative phosphorylation-related pathways and PPAR-Lipid synthesis-related pathways in the KEGG pathway enrichment analysis (Fig. 5B), and also related to the response to mis-folded proteins, nucleoside diphosphatase activity and polarized growth sites by GO analysis (Fig. 5C). In addition, they were mainly located in the cytoplasm and nucleus (Fig. 5D). These results indicate that GIM may also influence the development of GC by these proteins.

### Batch correction of GEO datasets and identification of critical genes of GIM

**Fig. 6:**
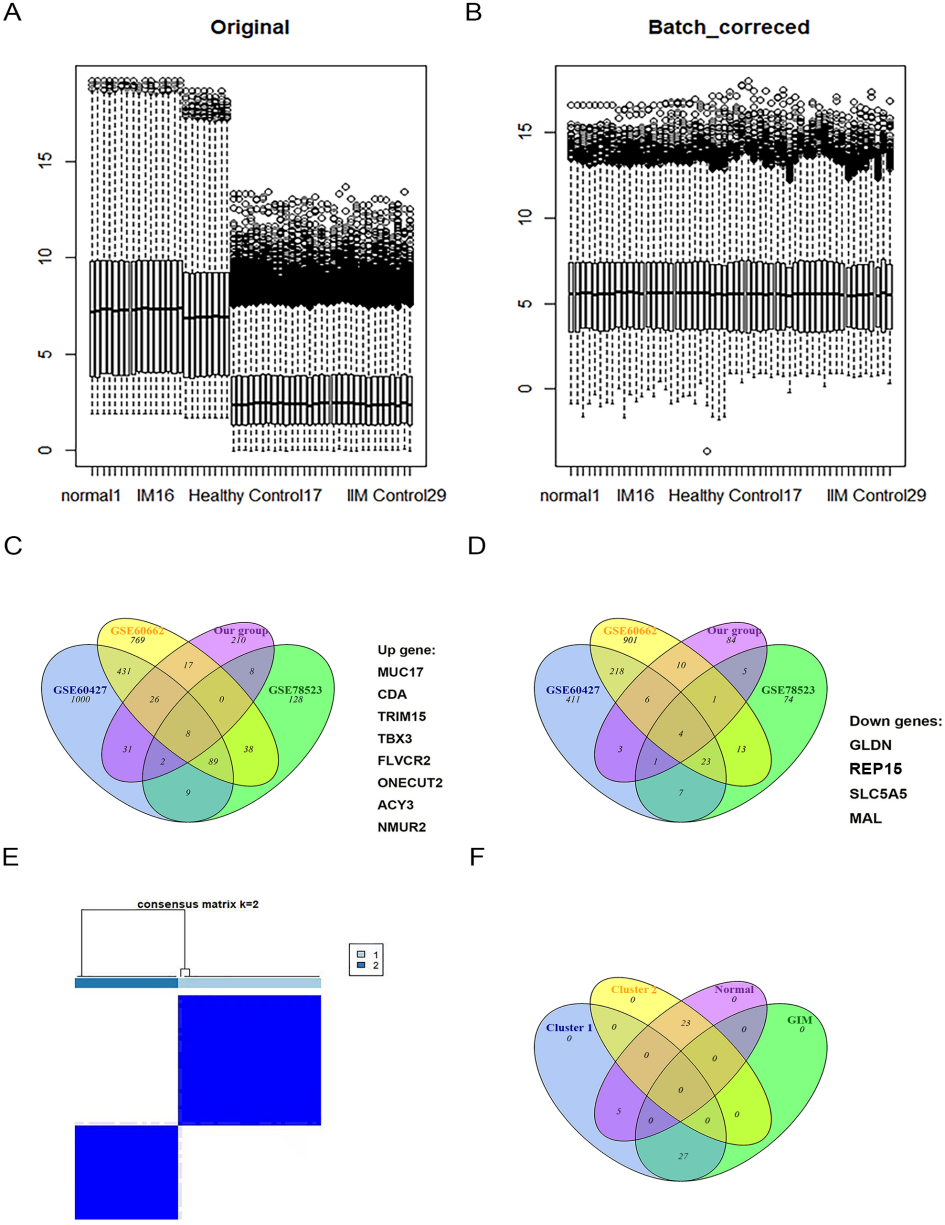
Batch correction between GEO datasets and identification of GIM key genes. (**A**&**B**) Batch correction between GEO datasets of GIM and normal samples. (**C**&**D**) The intersection of the upregulated and downregulated DEGs in the GIM sample from GEO datasets and the experimental group. (**E**) Cluster analysis of samples among GEO datasets. (**F**) The intersection of two clusters with GIM and normal samples in the GEO database

To better make use of the GIM and normal samples in the GEO datasets and screen for potential markers of GIM, batch effects were removed among GSE60427, GSE60662 and GSE78523 as GIM train datasets (Fig. 6A&B). The data visualized by Venn diagrams allowed us to determine the intersection of the GEO datasets DEGs and the experimental group DEGs. The essential genes, *MUC17*, *CDA*, *TRIM15*, *TBX3*, *FLVCR2*, *ONECUT2*, *ACY3*, and *NMUR2*, were upregulated in GIM samples compared with normal samples; the down-regulated essential genes were *GLDN*, *REP15*, *SLC5A5*, and *MAL* (Fig. 6C&D). The *P* value of these genes was processed multiple testing corrections in Supplementary Table 3. In addition, GSEA enrichment analysis (Supplementary Fig. 5) was conducted on the 13 genes exhibiting differential expression between GIM and normal samples. It was observed that genes demonstrating elevated expression levels in GIM samples were primarily associated with cellular-cytokine interactions and immune-related functionalities. The samples from the above datasets were clustered into “Cluster1” and “Cluster2” according to these critical genes (Fig. 6E). “Cluster1” and “Cluster2” were intersected with the GIM samples and normal samples in the GEO databases by using a Venn diagram (Fig. 6F). Only 5 samples cannot be accurately divided into GIM and normal samples. The error rate is 5/55 = 9%<10%. Furthermore, the heatmap results also showed that these critical genes could be used to differentiate GIM samples from normal ones (Supplementary Fig. 6), suggesting that these genes could serve as potential markers for GIM. The analysis of the GIM test datasets revealed that the expression levels of *CDA*, *TBX3*, *FLVCR2*, *ONECUT2*, *ACY3*, and *NMUR2* were significantly elevated in the GIM samples compared with the normal ones. Conversely, the expression of *GLDN*, *REP15*, and *MAL* showed significantly reduced in GIM patients (Supplementary Fig. 7, *P* < 0.05). These findings validate prior research and underscore a high level of conformity in the expression patterns of GIM biomarkers.

### Validation of crucial genes linked to GIM

**Fig. 7:**
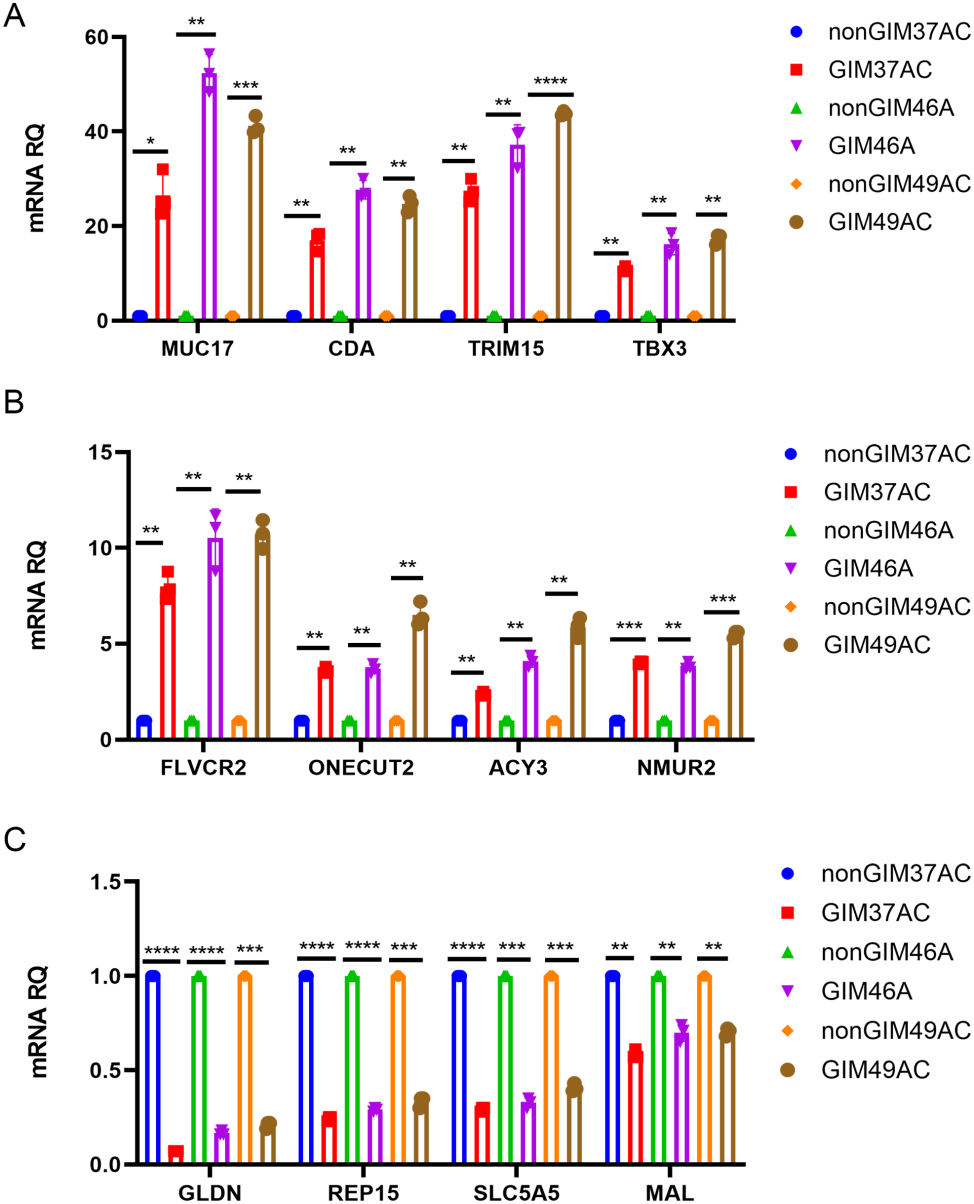
qRT-PCR validation of key GIM genes. (**A**&**B**) The key upregulated genes in GIM. (**C**) The key down-regulated key genes in GIM. *: *P*<0.05; **: *P*<0.01; ***: *P*<0.001; ****: *P*<0.0001

Based on qRT-PCR, we found that compared with normal samples, the expression of *MUC17*, *CDA*, *TRIM15*, *TBX3*, *FLVCR2*, *ONECUT2*, *ACY3*, and *NMUR2* was significantly (*P*<0.05) higher in GIM (Fig. 7A&B), while expression of *GLDN*, *REP15*, *SLC5A5*, and *MAL* was significantly lower (*P* < 0.05, Fig. 7).

## Discussion

This work confirmed that the ALI model provides the possibility for constructing GIM cells in vitro. We cultured human GIM and gastric mucosal epithelial cells using the ALI model for the first time, detected *MUC5AC* expression in normal gastric epithelium samples and *MUC2* expression in GIM samples by IF, and verified the long-term stability of the GIM-ALI model for expressing intestinal markers, epithelial stem markers, stem markers and gland cell type markers using qRT-PCR. Consistent with our results, *MUC5AC* is secreted by the pit cells of the gastric antrum and corpus [67], and *MUC2* is a gut marker highly expressed in GIM [68]. Studies had shown that *CDX1*, *CDX2*, and *MUC2* are specifically expressed in GIM [69]; markers of epithelial stem include *CD44*, *LGR5* and *CTNNB* [70–72], and *KRT18*, *KRT19* and *CDH1* are markers of epithelial cells [73–75]. Gastric gland cell type markers include *PGC*, *MUC5AC*, *MUC6*, and *ATP4B*. *PGC* is considered the final product of gastric mucosa maturity and differentiation [76]. The marker of gastric pit cells is *MUC5AC*, and the marker of gastric pyloric cells is *MUC6* [77]; *CHGA* protein secreted by gastric mucosal enterochromaffin-like (ECL) cells is an acidic protein [78]. The protein expressed by *ATP4B* is a proton pump that plays a crucial role in gastric acid secretion [79]. Thus, adopting these selected genes to verify the stability of the GIM-ALI model in this study provided a proof-of-concept, representing a reliable and useful strategy for the future.

Transcriptomic and MS analyses were performed on human GIM and normal samples. Oxidative phosphorylation, an important source of energy and metabolic precursors in GC cells, provides a potential target for GC therapy [80]. The highly expressed protein in GIM samples was primarily associated with oxidative phosphorylation-related pathways, which suggests that these pathways may be necessary for GIM to develop into GC. By combining the transcriptomic analysis results of the human GIM and normal samples in the GEO datasets with the results in the experimental group, potential markers of GIM were: *MUC17*, *CDA*, *TRIM15*, *TBX3*, *FLVCR2*, *ONECUT2*, *ACY3*, *NMUR2*, *GLDN*, *REP15*, *SLC5A5*, and *MAL*. After verification by qRT-PCR experiments, the differences were all statistically significant (*P*<0.05).

Among the potential markers of GIM found in this study, only *ONECUT2* has been reported in human GIM samples, which can induce and trigger the expression of *ACSL5* through epigenetic changes in GIM. *ONECUT2* and *ACSL5* may synergistically promote the transdifferentiation of gastric mucosal epithelial cells to GIM cells and the progression of GIM to GC [81]. In GC, the expression of *TRIM15* and *TBX3* are specific and independent factors for poor prognosis [82, 83]. The suppression of GC cells invasion and metastasis is accomplished through the inhibition of STAT3 phosphorylation by MAL [84]. This supports the idea that the upregulation of these genes, *TRIM15*, *TBX3*, and *MAL*, are potentially responsible for the occurrence of GIM and GC.

Currently, stimulation of GES-1 cells by bile acid induces the GIM phenotype, which contains certain limitations, as the model solely exhibits an elevation in GIM markers through in vitro intervention [85]. Furthermore, GIM features can be induced by overexpressing *CDX2* [85] or knocking out *ATP4a* [86] in the gastric mucosa of mice, albeit this model, utilizing mice and employing gene-editing technology to induce the emergence of the GIM phenotype, differs in species from humans. Furthermore, human induced pluripotent stem cells (hiPSCs) were differentiated into gastric organoids, subsequently in which overexpressed *CDX2* via the tet-on system, the expression of partly gut genes and previously reported genes associated with GIM were enhanced. Despite the utilization of hiPSCs, this model is also gene-edited as well as incapable of collecting mucus secreted by human GIM goblet cells [18]. None of the three models featuring GIM phenotypes in the current study can realistically portray the state of human GIM.

Moreover, the GIM-ALI model represents a significant advancement, allowing for the long-term, stable in vitro cultivation of GIM cells. This achievement fills a critical void in the realm of in vitro GIM cell models, paving the way for enhanced biomarker discovery and more in-depth investigations into the mechanisms underlying GIM. Notably, this model offers a unique avenue for the collection of mucus secretions above the inserts, a distinguishing feature of GIM cells [87], thereby facilitating a deeper exploration of the role of mucus in GIM pathogenesis. In parallel, the GIM-ALI model presents novel strategies for investigating the impacts of diverse substances, encompassing drugs, metabolites, and bacteria co-cultivated with GIM cells treated under the insert, as well as for finding out the interactions of GIM cells with other cellular entities. The GIM-ALI model offers a novel framework, facilitating a deeper exploration of the pathogenic factors associated with the onset of GIM and elucidating how cytokines secreted by other cells influence the initiation and progression of GIM [16]. These attributes render the GIM-ALI model to simulate the real situation of GIM tissues in vivo. This provides a valuable tool for unraveling the mechanisms by which these agents either hinder or promote GIM progression.

While the GIM-ALI model presents certain advantages over conventional GIM in vitro models and in vivo animal models, it still harbors limitations. Primarily, the model solely comprises the GIM epithelial layer, thus lacking engagement with the vascular, immune, and nervous systems [88]. Moreover, the GIM-ALI models are relatively high costs attributable to the necessity for reagents and growth factors/inhibitors, alongside their reliance on extracellular matrices (e.g., matrix) and prolonged culture periods [89]. In addition, a significant amount of time is required for GIM-ALI model cultivation and the present GIM-ALI model fails to faithfully replicate the metabolism of its parent organ. To mitigate certain constraints, efforts are being made by bioengineers to develop precisely defined culture media and extracellular matrix systems [90]. Notably, a gastric epithelial ALI model has been developed by the German laboratory, and the identical GIM-ALI model has been established using our methodology for drug screening purposes (data in process).

Briefly, the development of the GIM-ALI model is a significant advancement, offering a novel in vitro platform for GIM studies. This model’s ability to mimic in vivo conditions and facilitate mucus secretion studies is particularly noteworthy. In addition, the identification of novel potential markers of GIM and the innovative GIM-ALI model represent significant contributions to the field, offering a new scene for research and therapeutic intervention. Our findings have significant implications for understanding GIM pathogenesis and its progression to GC, offering potential biomarkers for early detection and targets for therapeutic intervention [91]. In the future, our research aims to leverage the GIM-ALI model for crucial applications, including drug screening and the development of innovative therapeutic interventions. We also plan to delve into genetic and epigenetic studies to unravel the intricate mechanisms underlying GIM progression. Moreover, we envision adapting this model to individual patient cells to explore its potential in personalized medicine approaches. Furthermore, we are committed to expanding the utility of the GIM-ALI model in various aspects of gastrointestinal tract research. This includes investigating other precancerous lesions and conducting direct comparisons with in vivo observations to gain deeper insights. We are also dedicated to overcoming technical challenges such as batch effects and sample diversity that may arise during experimentation. To bolster the credibility and widespread adoption of the GIM-ALI model, we will actively collaborate with the broader research community. By engaging multiple research centers, we aim to assess and validate the model’s performance across different settings. This collaborative effort will enhance the reliability and generalizability of our findings.

## Conclusion

GIM cells using the ALI model were successfully cultured for the first time, revealing 12 potential markers of GIM. This study fills the gap of lacking effective in vitro models of human GIM cells and provides insights into the GIM pathogenesis. It’s recommended that future studies explore the identified markers’ therapeutic potential, expand the GIM-ALI model’s applications, and strive for larger, more diverse study cohorts to validate and extend these findings.

### Electronic supplementary material

Below is the link to the electronic supplementary material.

Supplementary Material 1

Supplementary Material 2

Supplementary Material 3

Supplementary Material 4

## Data Availability

The data used in this study were from the patients with GIM and the GEO (https://www.ncbi.nlm.nih.gov/geo/) database. Our analyses’ protocols and raw figures or other information related to our study could be asked from the corresponding author on reasonable request.

## Abbreviations

CAG: Chronic atrophic gastritis
GIM: Gastric intestinal metaplasia
GC: Gastric cancer
GO: Gene Ontology
KEGG: Kyoto Encyclopedia of Genes and Genomes
DEGs: Differentially expressed genes
DEPs: Differentially expressed proteins
PPI: Protein-protein interaction
GEO: Gene Expression Omnibus
