# Supplementary material for: Revealing the pathogenesis of gastric intestinal metaplasia based on the mucosoid air-liquid interface
Source: J Transl Med. 2024 May 17;22:468. doi: 10.1186/s12967-024-05276-7 (PMC11101349; doi:10.1186/s12967-024-05276-7)
Supplement: Supplementary file 4 — Supplementary Material 4 [file 12967_2024_5276_MOESM4_ESM.docx]

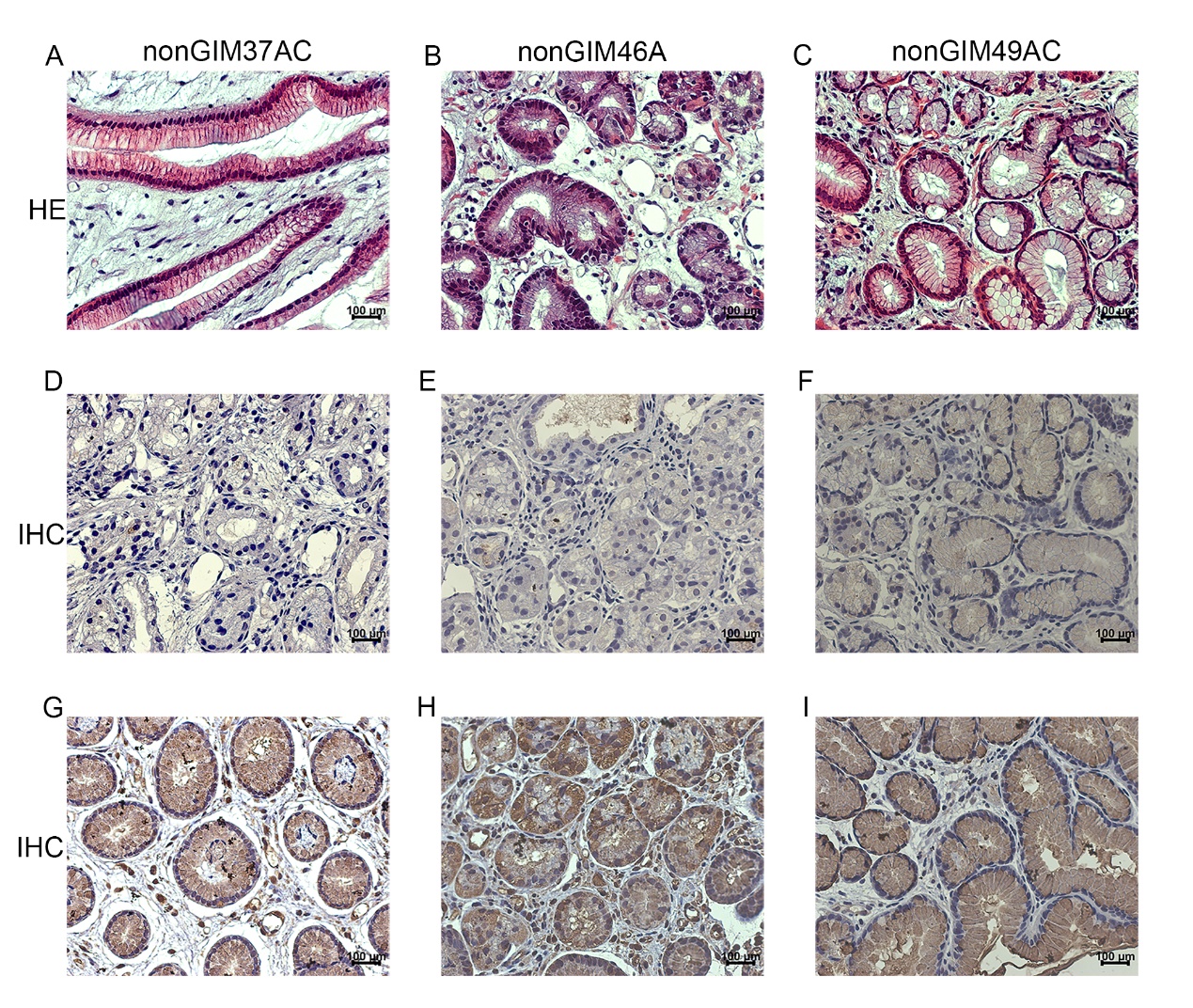


**Supplementary Figure 1. Identification of the normal gastric organization.** (A-C) HE staining of 3 normal gastric samples. The nucleus is stained blue and the cytoplasm is stained red. (C-F) Immunohistochemistry of 3 normal gastric samples. Nuclei are stained blue and *MUC2* is stained brown. (G-I) Immunohistochemistry of 3 normal gastric samples. Nuclei are stained blue and *MUC5AC* is stained brown.


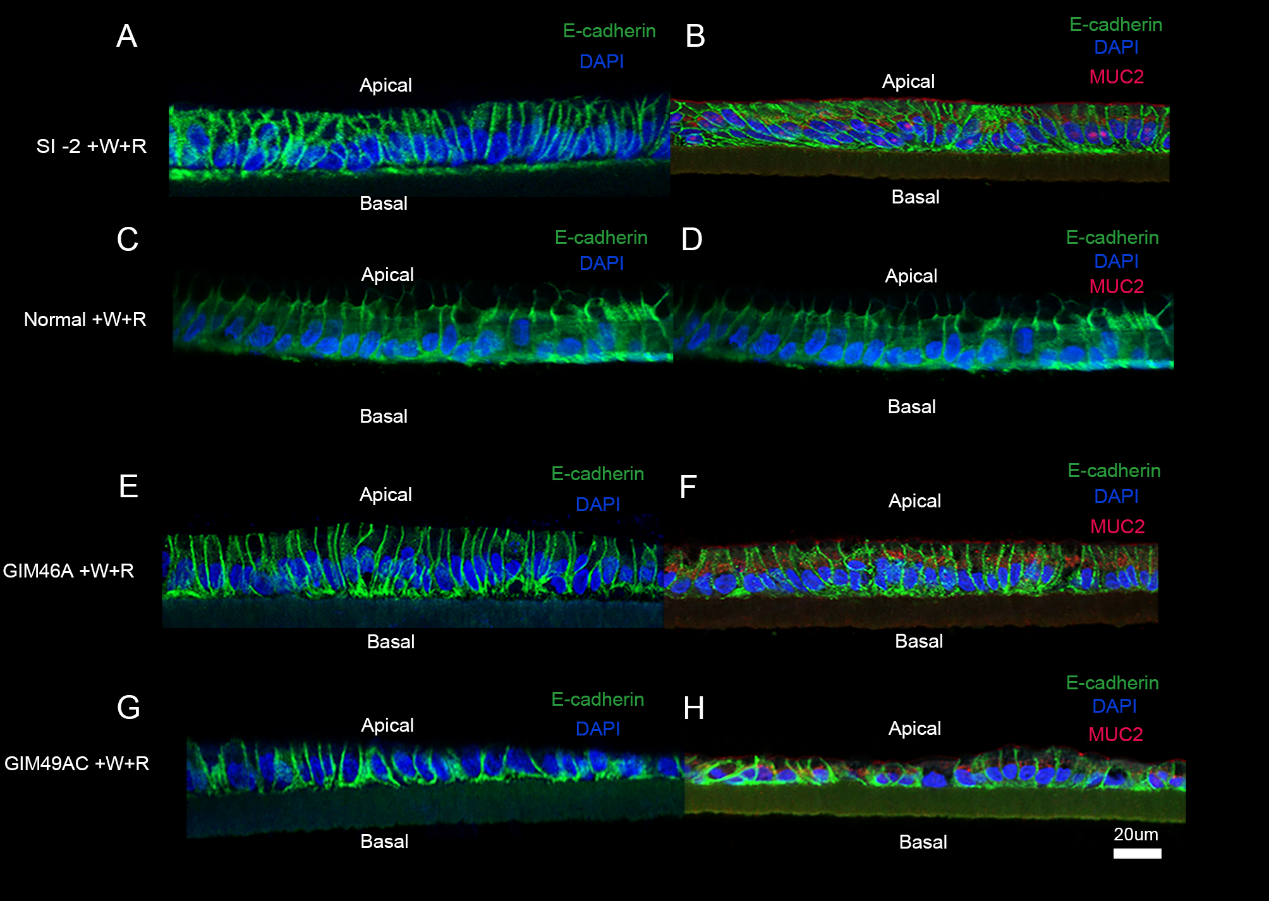


**Supplementary Figure 2. The expression of *MUC2* in small intestine (A&B), normal gastric epithelium (C&D) and GIM (E-H) samples. *E-cadherin* is depicted in green, DAPI in blue, and *MUC2* in red. The intensity of the color indicates the protein's expression level, with brighter shades corresponding to higher expression.**


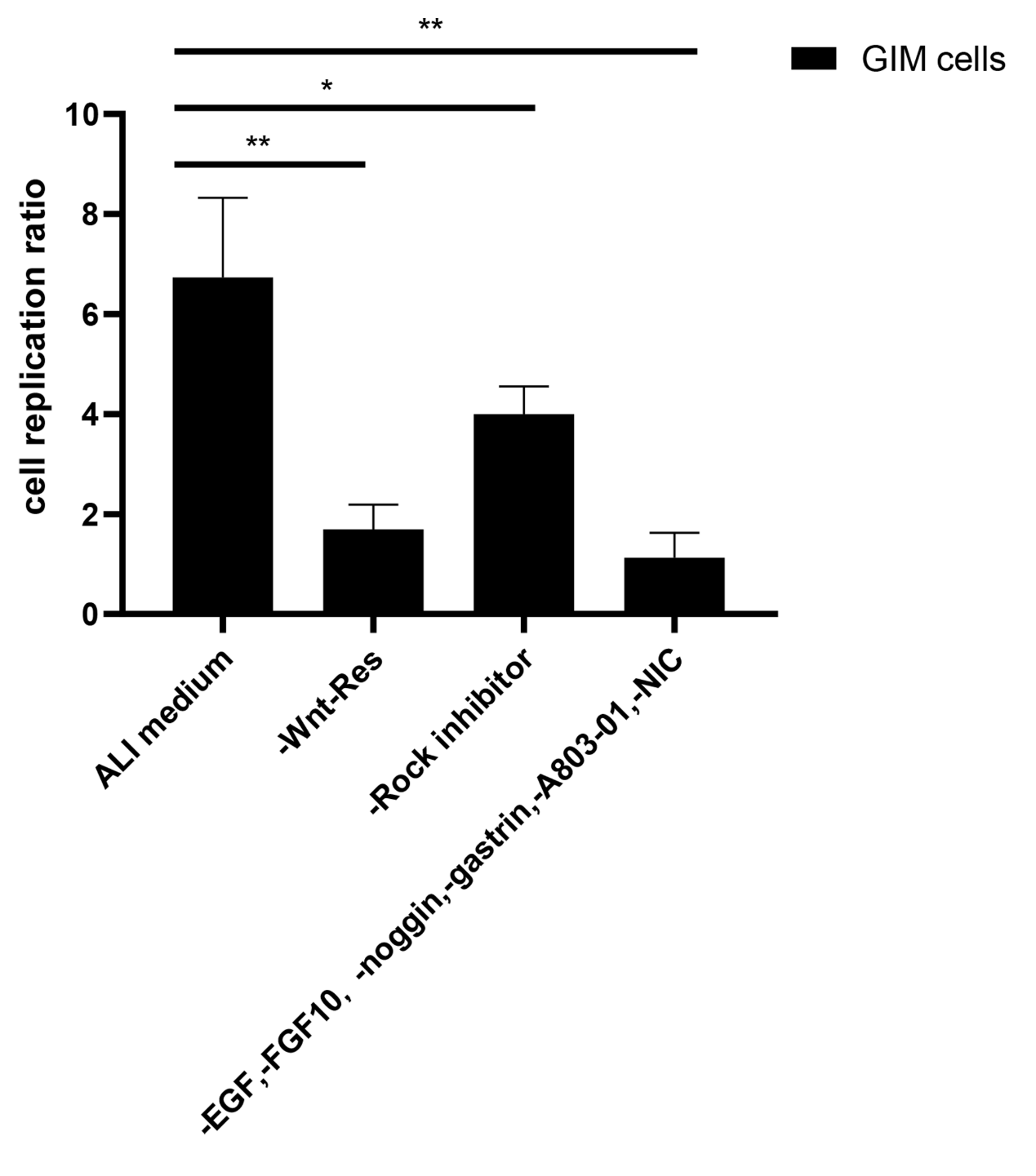


**Supplementary Figure 3. The proliferation of cells cultured in a medium with different factors. *: *P*<0.05; **: *P*<0.01.**

**
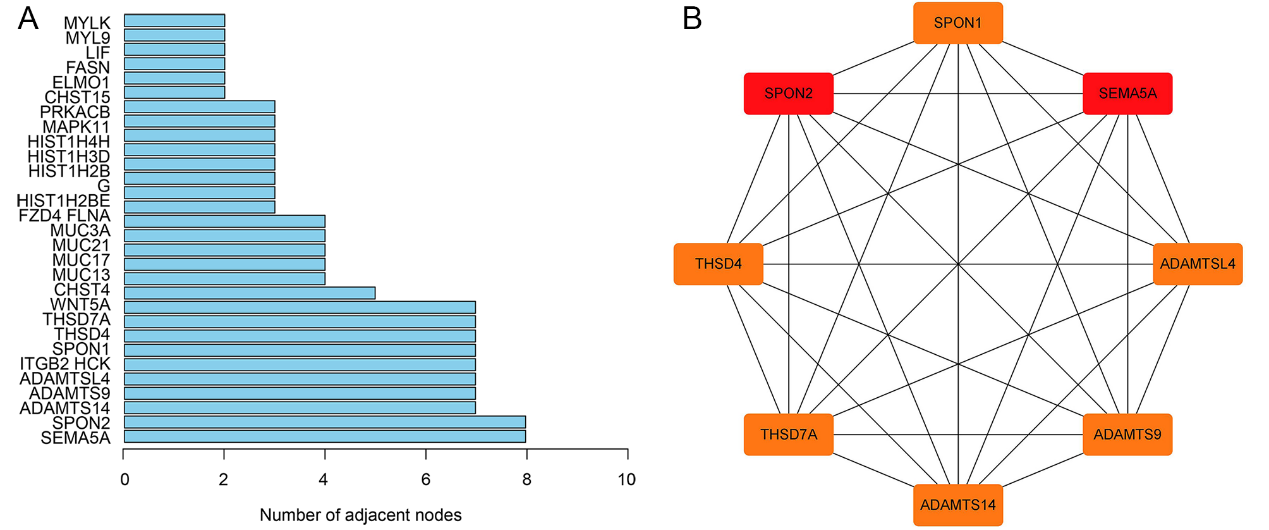
**

**Supplementary Figure 4. The differentially expressed genes (DEGs) contacts between** **GIM and normal samples.** (A) Bar plot of the top 30 DEGs most connected to adjacent nodes. (B) PPI network diagram of the top 8 most connected DEGs.


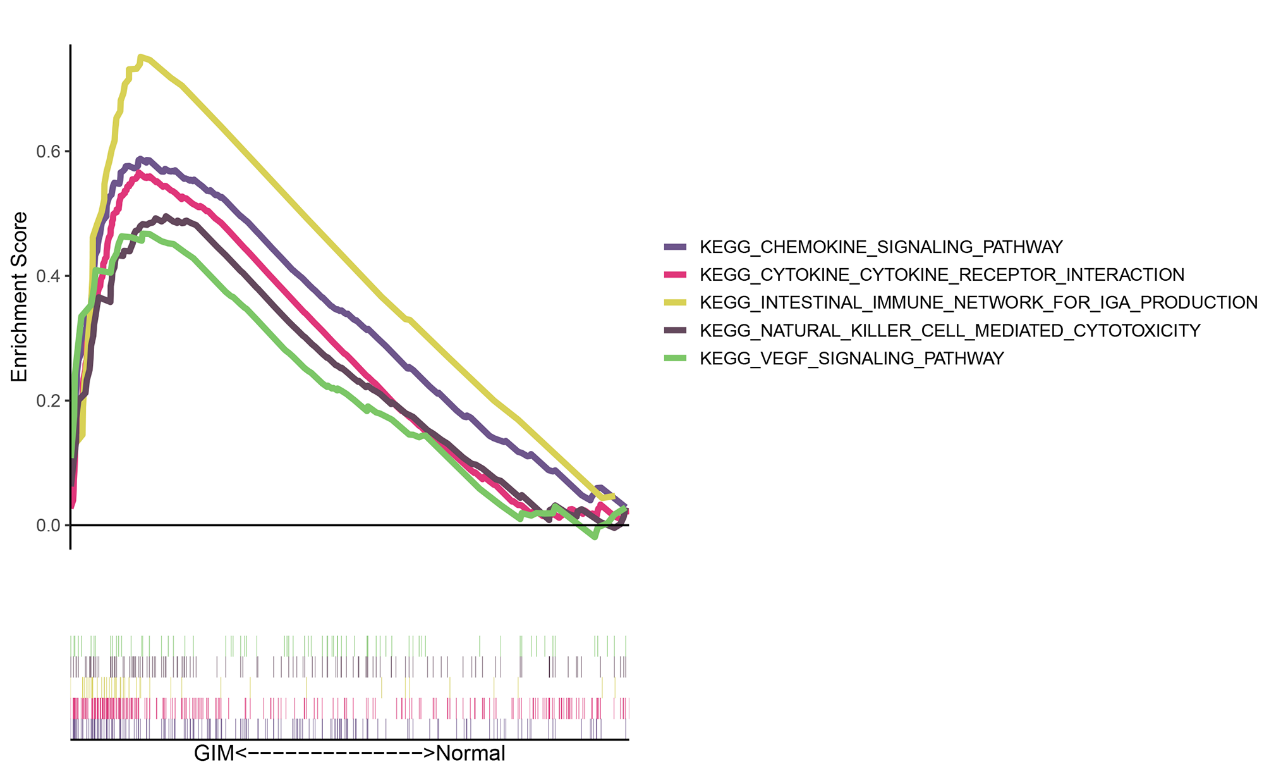


**Supplementary Figure 5. GESA enrichment analysis of key genes upregulated in GIM samples.**

**
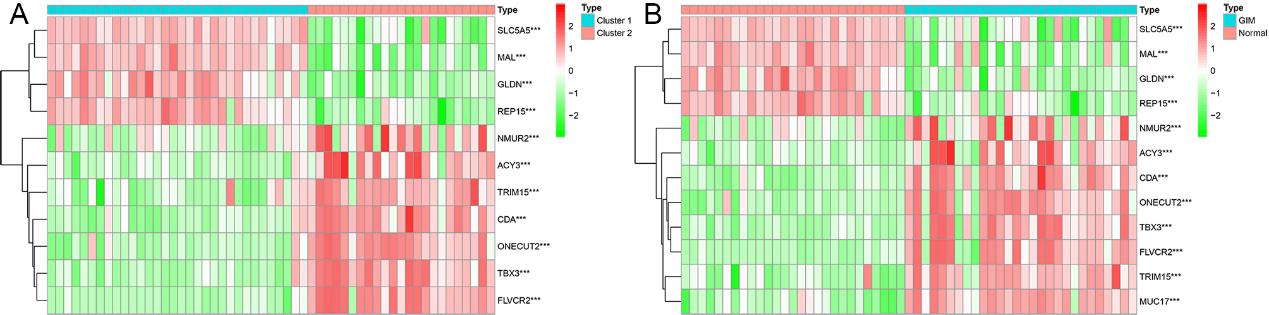
**

**Supplementary Figure 6. The differences between the two clusters (A) and the GIM and normal samples (B) in the GEO databases were compared in the form of heat maps according to the expression of key genes of GIM. Green represents low expression, white represents intermediate expression, and red represents high expression.**

**
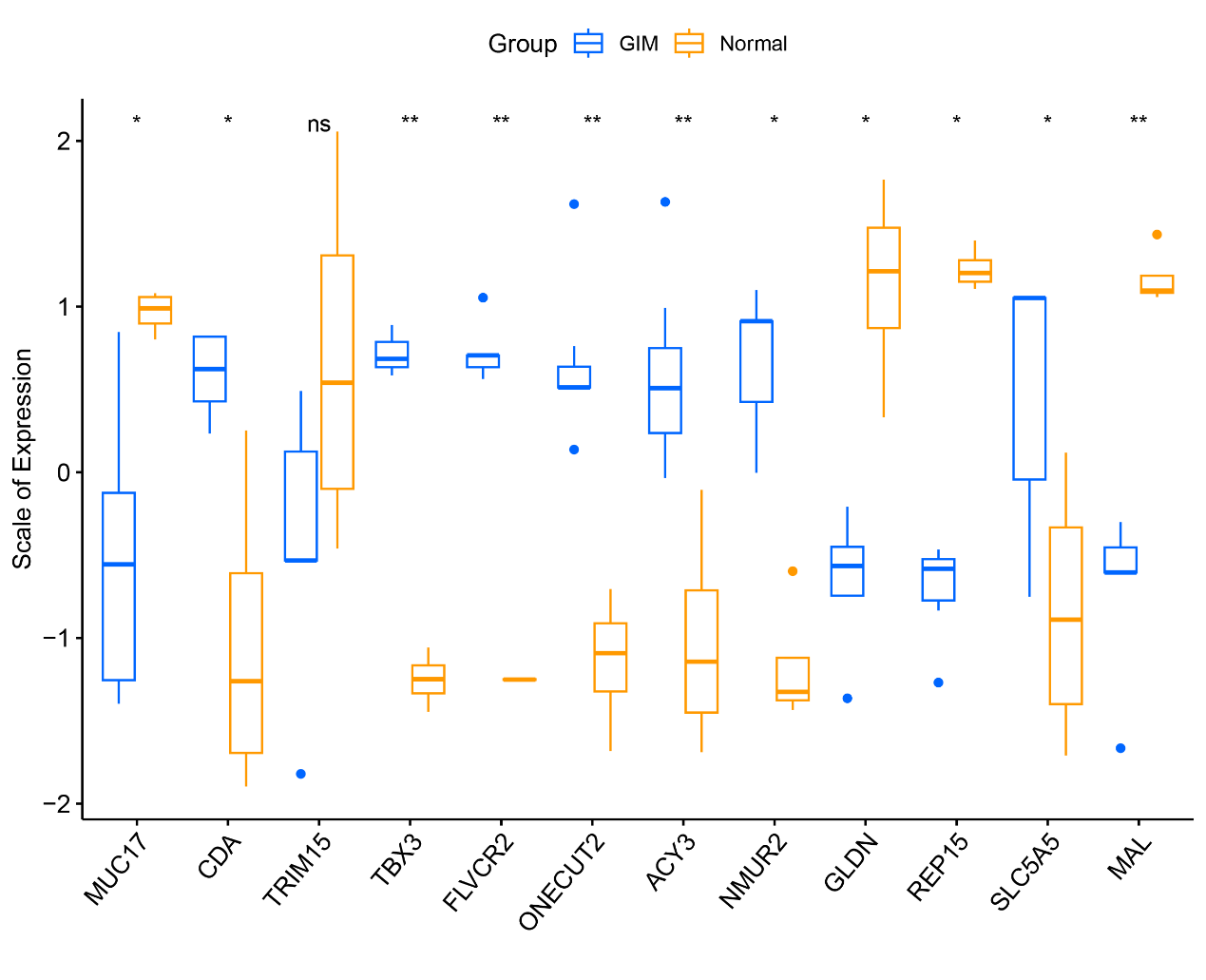
**

**S****upplementary Figure 7. The expression of GIM biomarkers from GIM test datasets. ns: no sense； *: *P*<0.05; **: *P*<0.01.**

**Supplementary Table 1. GIM patient information.**

| Name | Age | Gender | *Hp* infection level | GIM severity |
| --- | --- | --- | --- | --- |
| GIM37AC | 50 | Male | None | Severe |
| GIM46A | 43 | Male | *Hp*(+++) | Mild |
| GIM49AC | 37 | Female | *Hp*(+) | Moderate |

**Supplementary Table 2.** qRT-PCR primer sequences for the targeted genes.

| Primers | **Forward Sequence** | **Reverse Sequence** |
| --- | --- | --- |
| GAPDH | ACAACTTTGGTATCGTGGAAGG | GCCATCACGCCACAGTTTC |
| CDX1 | GGTGGCAGCGGTAAGACTC | TGTAACGGCTGTAATGAAACTCC |
| CDX2  MUC6  MUC5AC  MUC2  LGR5  LRIG1  ITLN1  TFF3  CD44  CTNNB  KRT8  KRT18  KRT19  PGC  ATP4B  OLFM4  CHGA  MUC17  CDA  TRIM15  TBX3  FLVCR2  ONECUT2  ACY3  NMUR2  MAL2  GLDN  MALAT1  SLC5A5  CDH1  MAL | GACGTGAGCATGTACCCTAGC  CAGCTCAACAAGGTGTGTGC  CTTCTCAACGTTTGACGGGAAGC  GAGGGCAGAACCCGAAACC  CTCCCAGGTCTGGTGTGTTG  CTCGCCTTGCCTTCTCCTTC  AACACTGAGCACCACTGCAT  CTGCAGGAAGCAGAATGCAC  CTGCCGCTTTGCAGGTGTA  AAAGCGGCTGTTAGTCACTGG  CAGAAGTCCTACAAGGTGTCCA  GGCATCCAGAACGAGAAGGAG  AACGGCGAGCTAGAGGTGA  AGTCTATCCGTGAGACCATGAA  TGGGTGTGGATCAGCCTGTA  ACCTTTCCCGTGGACAGAGT  CGCTGTCCTGGCTCTTCTG  TCTCAGCACGTTAGGACAGGT  AAGTCAGCCTACTGCCCCTAC  TCCCTGAAGGTGGTCCATGAG  GAGGCTAAAGAACTTTGGGATC  CCCTGAGCTATGCCTTGACC  GGAATCCAAAACCGTGGAGTAA  TGGGCACCTGCTTAATCGC  AGATGTGGCGCAACTACCC  GTCCGTGACAGCGTTTTTCTT  TGGTGCCGATCCGAGTGAT  GAGCAAAGGAAGTGGCTTA  CCTATCGCTATGGCCTCAAGT  CGAGAGCTACACGTTCACGG  TCACCTTGGACGCAGCCTA | GCGTAGCCATTCCAGTCCT  TGGGGAAAGGTCTCCTCGTA  CTTGATCACCACCACCGTCTG  GGCGAAGTTGTAGTCGCAGAG  GCTCGCAATGACAGTGTGTG  TAACTCAGGTTTAGGCTCCG  GGGTTCCCTCCCACAAAACT  CGGGAGCAAAGGGACAGAAA  CATTGTGGGCAAGGTGCTATT  CGAGTCATTGCATACTGTCCAT  CTCTGGTTGACCGTAACTGCG  ATTGTCCACAGTATTTGCGAAGA  GGATGGTCGTGTAGTAGTGGC  GCGGTACTTCCAAGCAGGA  CTGGTCTTGGTAGTCCGGTG  TGGACATATTCCCTCACTTTGGA  TCACCTCGGTATCCCCTTTATTC  TCGAGGTCATCTCAGGGTTGG  GATAGCGGTCCGTTCAGCAC  CAGGATCTTGCCCGAGGATT  CATTTCGGGGTCGGCCTTA  ATCACCATGCGATTCAGAAGAG  CTCTTTGCGTTTGCACGCTG  AGGGTCCTCATCCTTGAGAAAAT  CGAAGCACACGGTCTCAAAGA  AATTGAGGCTGCTACGTTTATG  GGCTGTCCATCCAATCCGTT  TCTTCAAGAGAGATATTTAA  CGTGGCTACAATGTACTGCAAA  GGGTGTCGAGGGAAAAATAGG  GAAGCCGTCTTGCATCGTGAT |

**Supplementary Table 3. The multiple testing correction of *P* value of GIM essential genes.**

| Symbol | *P* value | Bonferroni | BH | Holm | Hochberg | Hommel | BY |
| --- | --- | --- | --- | --- | --- | --- | --- |
| ONECUT2 | 1.04E-09 | 1.35E-08 | 1.35E-08 | 1.35E-08 | 1.35E-08 | 1.35E-08 | 4.30E-08 |
| TBX3 | 9.38E-08 | 1.22E-06 | 6.10E-07 | 1.13E-06 | 1.13E-06 | 1.13E-06 | 1.94E-06 |
| NMUR2 | 3.06E-06 | 3.98E-05 | 1.33E-05 | 3.37E-05 | 3.37E-05 | 3.37E-05 | 4.22E-05 |
| MUC17 | 7.81E-06 | 0.000102 | 2.54E-05 | 7.81E-05 | 7.81E-05 | 7.81E-05 | 8.07E-05 |
| GLDN | 0.000224 | 0.002915 | 0.000559 | 0.002018 | 0.002018 | 0.001505 | 0.001777 |
| ACY3 | 0.000258 | 0.003352 | 0.000559 | 0.002063 | 0.002063 | 0.001547 | 0.001777 |
| FLVCR2 | 0.000595 | 0.007736 | 0.000969 | 0.004165 | 0.002251 | 0.00186 | 0.00308 |
| CDA | 0.000734 | 0.009545 | 0.001061 | 0.004165 | 0.002251 | 0.002092 | 0.003373 |
| TRIM15 | 0.00086 | 0.011177 | 0.001118 | 0.004165 | 0.002251 | 0.002092 | 0.003555 |
| SLC5A5 | 0.001119 | 0.014552 | 0.001323 | 0.004165 | 0.002251 | 0.002239 | 0.004207 |
| MAL | 0.001395 | 0.01813 | 0.001511 | 0.004165 | 0.002251 | 0.002251 | 0.004805 |
| MALAT1 | 0.002251 | 0.029263 | 0.002251 | 0.004165 | 0.002251 | 0.002251 | 0.007158 |
